# Biocompatible Ag/Fe-Enhanced TiO_2_ Nanoparticles as an Effective Compound in Sunscreens

**DOI:** 10.3390/nano10030570

**Published:** 2020-03-21

**Authors:** Adrian Ionut Nicoara, Vladimir Lucian Ene, Bianca Beatrice Voicu, Mihaela Adriana Bucur, Ionela Andreea Neacsu, Bogdan Stefan Vasile, Florin Iordache

**Affiliations:** 1 Faculty of Applied Chemistry and Materials Science, Department of Science and Engineering of Oxide, Materials and Nanomaterials, University Politehnica of Bucharest, 060042 Bucharest, Romania; adrian.nicoara@upb.ro (A.I.N.); beatrice.voicu@stud.fim.upb.ro (B.B.V.); mihaela.bucur1302@stud.chimie.upb.ro (M.A.B.); neacsu.a.ionela@gmail.com (I.A.N.); bogdan.vasile@upb.ro (B.S.V.); 2 National Centre for Micro and Nanomaterials, University Politehnica of Bucharest, 060042 Bucharest, Romania; 3 Faculty of Veterinary Medicine, Department of Biochemistry, University of Agronomic Science and Veterinary Medicine, 011464 Bucharest, Romania; floriniordache84@yahoo.com

**Keywords:** Ag/Fe-enhanced TiO_2_ powders, sunscreens, UV protection, sol-gel synthesis, microwave-assisted hydrothermal synthesis, sun protection factor

## Abstract

In this work, valuable biocompatible Ag/Fe-enhanced TiO_2_ nanoparticles are comparatively prepared by a conventional wet chemistry method (sol-gel) and a rapid, efficient, hybrid unconventional method (microwave-assisted hydrothermal synthesis). In order to establish their application as effective compounds in sunscreens, the obtained powders were first structurally and morphologically characterized, analyses from which their nanodimensional character, crystalline structure and thermal behavior were highlighted. The evaluation of sunscreen effectiveness is based on the determination of the sun protection factor (SPF). It was observed that silver enhancing increases the SPF significantly, especially when compared to the pristine samples. The obtained Ag/Fe-enhanced TiO_2_ powders were also evaluated from the point of view of their biocompatibility on amniotic fluid stem cells, and the results indicated an enhance of cell proliferation when exposed to the synthesized nanostructures.

## 1. Introduction

Throughout life, there are many factors that can lead to skin degradation, but the most common one is the exposure to ultraviolet radiation (UV). While UVC radiation (200–290 nm wavelength) is absorbed by the ozone layer, both UVB (290–320 nm wavelength) and UVA (320–400 nm wavelength) radiation are well identified as contributors to various molecular events that facilitate skin carcinogenesis and eventually lead to skin cell injury, melanoma and non-melanoma skin cancer [1,2,3]. The detrimental effects of UV radiation on epidermal cells consist in DNA alteration and reactive oxygen species (ROS) formation, which later interact with other molecules, triggering oxidative harm. The UV exposure also amplifies nitric oxide (NO) concentrations, that in high levels becomes detrimental to the human body and interacts as a free radical with ROS to produce additional NO end-products [4,5]. 

To prevent these adverse effects of UV radiation, scientists have introduced sunscreen creams. The effectiveness of a sunscreen cream is given by the SPF (sunscreen protection factor), determined by the ratio of MED (minimum dose of erythema) on protected skin, over MED on unprotected skin [6]. Currently, there are two types of used sunscreens: (i) Those that contain physical (inorganic) blockers that absorb, scatter and reflect UV rays; and (ii) chemical blockers, meaning those organic compounds that absorb UV radiation. Even though organic filters (aminobenzoic acid, avobenzone, octocrylene, oxybenzone) are widely used in sunscreens, they produce a series of unwanted reactions due to their ability to penetrate the skin, influence hormonal activity and generate chemical reactions at skin level [7]. 

Lately, inorganic UV filters such as titanium dioxide (TiO_2_) or zinc oxide (ZnO) have been extensively used, especially in products for children and people with sensitive skin, mainly because of their low potential to cause irritation [8]. TiO_2_ has attracted the interest of many researchers in the field of materials science, due to its unique combination of electrical, optical, photo-catalytic properties etc. TiO_2_ is a semiconductor with large band gap (3.0–3.2 eV), high refractive index and transparency in the visible field [9,10,11]. Titanium dioxide presents three stable polymorphic forms: rutile (tetragonal), anatase (tetragonal) and brookite (orthorhombic). The first two polymorphic forms are studied because the anatase/rutile crystallization type plays a decisive role in the spectrum of titanium dioxide applications [12,13]. While being the least abundant of the three forms, the anatase is the most researched at laboratory scale. The brookite has been, until recently, less researched. All forms have been studied for applications in photocatalysis and photoelectrochemistry. At lower temperatures, anatase and brookite are more stable, but at elevated temperatures, rutile is the stable form [14]. 

Yet, some drawbacks of TiO_2_—such as high band gap energy, small adsorption capacity, problematic phase separation and high recombination of electron–hole pairs—constrains its applicability. In order to surpass these limitations and expand the range of light absorption spectra (not only UV) or the photocatalytic properties of TiO_2_, numerous approaches, such as element-enhancing, facet modification, etc., have been recently proposed [15,16,17]. Enhancing with metals such as Fe, Au, Ag, etc., is an encouraging method to increase the photocatalytic activity by reducing the band gap of TiO_2_ while maintaining the integrity of the crystalline structure [18,19]. In general, ion enhancing contributes to the improvement of TiO_2_ photocatalytic properties in several ways: (i) By reducing the bandgap and encouraging the adsorption of the visible region of the solar spectrum (i.e., enhancing with N, S, C, B, etc.); (ii) by improving the conductivity of TiO_2_ and the mobility of charge carriers, the increased charge traps can reduce bulk recombination and separate photogenerated electrons and holes more efficiently (e.g., Zn, Fe, and Y); and (iii) altering the conduction band position of TiO_2_ with certain heterovalent or homovalent metal ion dopants, such as Zr^4+^, Nb^5+^, and W^6+^, further affects the carrier transfer properties [11,20]. When adding noble metal nanoparticles, additional energy levels are introduced into the energy band structure that can be used to capture electrons. Furthermore, creating gaps that separate the carriers from the bands, helps more carriers to diffuse to the surface, which could endorse separation of photo-generated electron-hole pairs due to the formation of Schottky barrier at TiO_2_-metal junctions [21,22].

Among these enhancing metal materials, Ag has a certain photocatalytic ability, some advantages of raw material preparation and cost, and it is considered to be a material that has a prospect of industrial application [23,24]. However, it was found that excessive enhancing with silver may decrease TiO_2_ photocatalytic activity because it occupies active sites on its surface [21]. When discussing about Fe-enhanced TiO_2_, several studies highlight the influence of iron atoms on the oxide, dependent of its crystal structure (i.e., production of more oxygen vacancies in anatase than in rutile), generally enhancing the photocatalytic activity of TiO_2_ powders and thin films [25,26,27]. 

In this work, valuable biocompatible Ag/Fe-enhanced TiO_2_ nanoparticles are comparatively prepared by a conventional wet chemistry method (sol-gel) and a rapid, efficient, hybrid unconventional method (microwave-assisted hydrothermal synthesis), and characterized as prospective effective compounds in sunscreens.

## 2. Materials and Methods 

### 2.1. Materials 

The Ag/Fe-enhanced TiO_2_ samples were obtained by microwave-assisted hydrothermal and sol-gel methods. The chemical reagents used were titanium tetraisopropoxide (TTIP, Ti(OCH-(CH_3_)_2_), 97%, Sigma-Aldrich), silver nitrate (AgNO_3_, >99%), iron chloride (FeCl_3_, >99%), nitric acid (HNO_3_, 65%), isopropanol (C_3_H_8_O, >99.7%), purchased from Sigma-Aldrich, Darmstadt, Germany, and distilled water.

### 2.2. Sol-Gel Synthesis

For the sol-gel method, the TTIP:H_2_O:C_3_H_8_O = 1:155:1.5 ratio was used and 0.4% molar silver or iron precursors were added to obtain titanium dioxide particles enhanced with silver or iron ions. In order to obtain the anatase phase, the pH was adjusted to 2.5 by adding HNO_3_. The resulting solutions were stirred on a magnetic hob at 82.5 °C for 24 h at reflux (corresponding to the boiling temperature of the isopropanol), then centrifuged and washed with distilled water several times until obtaining a neutral pH, and dried in the oven at 45 °C for 72 h. 

### 2.3. Microwave-Assisted Hydrothermal Synthesis

For the microwave-assisted hydrothermal method the ratio TTIP:H_2_O = 1:100 was used. The general steps implied in this type of synthesis were previously described by Paduraru et al. [28]: Particularly, the precursors for Ti and Ag/Fe, along with distilled water and nitric acid, were weighed and transferred into Teflon vials. Afterwards, the vials (placed in a rack) were automatically pulled down into the reaction chamber, sealed and pre-pressurized with nitrogen. The obtained reaction mixtures were then subjected to a microwave-assisted hydrothermal treatment of 10 min, at 200 °C and the pressure of 20 bars, then cooled to room temperature. After the autoclaving process, the samples were centrifuged and washed several times with distilled water until obtaining a neutral pH and dried in the oven at 45 °C for 72 h. 

### 2.4. Structural and Morphological Characterization

In order to investigate the crystalline phases, the samples were analyzed using the PANalytical Empyrean equipment (from Malvern PANalytical, Bruno, Nederland) in Bragg–Brentano geometry equipped with a Cu anode (λ_CuKα_ = 1.541874 Å) X-ray tube. The X-ray diffractogram (XRD) was acquired in the range of 10°–80° 2θ angle, with an acquisition step of 0.02° and an acquisition time of 100 s per step. The scanning electron microscopy (SEM) images were performed with an Inspect F50 microscope coupled with an energy dispersive spectrometer (EDS) (Thermo Fisher—former FEI, Eindhoven, Nederland), while transmission electron microscopy (TEM) images were obtained using a Tecnai G2 F30 S-Twin microscope (Thermo Fisher—former FEI, Eindhoven, Nederland) equipped with scanning transmission electron microscopy (STEM)/ high-angle annular dark-field (HAADF) detector, EDS and energy filtered transmission electron microscopy (EFTEM)- electron energy loss spectroscopy (EELS) spectrometer. The Raman spectra were realized with a LabRAM HR Evolution instrument (from Horiba, Longjumeau, France), using a 633 nm LASER and 50× lens, while thermogravimetric (TG) analysis and differential thermal analysis (DTA) were performed using Shimadzu DTG-TA-50H equipment (from Shimadsu, Sanjo, Japan) at 20–900 °C.

### 2.5. Sun Protection Factor (SPF) Evaluation

For the determination of the SPF, the UV-VIS Evolution 300 Spectroscope (Thermo Fisher, Eindhoven, Nederland) was used, by performing a scan with a step of 5 nm on dispersions that contained 5% (wt) Ag/Fe-TiO_2_/TiO_2_ powder in distilled water. In order to determine the SPF of the obtained samples, Mansur’s relation (1) was used for wavelengths between 290 and 320 nm [29]:

(1) $$SPF_{spectrophotometric}=CF\times \overset{320}{\underset{290}{\sum }}EE\;\left(\lambda \right)\times I\;\left(\lambda \right)\times Abs\;\left(\lambda \right)$$

where *EE* is the erythemal effect spectrum; *I* represents the solar intensity; *Abs* is the absorbance of sunscreen product and *CF* represents the correction factor (= 10). The values of *EE* × *I* are constants and predetermined by Sayre et al. [30].

### 2.6. Biocompatiblity In-Vitro 

#### 2.6.1. Quantitative In-Vitro Evaluation of Biocompatibility—MTT Assay

The obtained Ag/Fe-enhanced TiO_2_ powders were first evaluated from the point of view of their biocompatibility by means of MTT (3-(4,5-dimethylthiazol-2-yl)-2,5-diphenyltetrazolium bromide) analysis, performed on mesenchymal stem cells isolated from amniotic fluid (AFSC). The cells were grown in Dulbecco’s modified Eagle’s medium (DMEM, Sigma-Aldrich, Missouri, MO, USA) supplemented with 10% fetal bovine serum and 1% antibiotics (penicillin and streptomycin) and changed twice a week. A dedicated MTT assay kit (Vybrant® MTT Cell Proliferation Assay Kit, Thermo Fischer Scientific, Massachusetts, MA, USA) was used. The analysis involves the cultivation of AFSC cells in 96-well plates, with a seeding density of 3000 cells/well, in the presence of the obtained Ag/Fe-enhanced TiO_2_ powders, for 72 h. Then 10 µL (12 mM) MTT was added and the cells were incubated at 37 °C for 4 h. Thereafter, 100 µL SDS-HCl solution (1 mg Sodium Dodecyl Sulphate + 10 mL HCl, 0.01 M) was added, followed by vigorous pipetting to solubilize the formed formazan crystals. The optical density (OD) of solubilized formazan reading was performed after 1 h using a TECAN Infinite M200 spectrophotometer (Männedorf, Switzerland) at 570 nm.

#### 2.6.2. Oxidative Stress Assessment—GSH-Glo Glutathione Assay 

For this analysis, AFSC were seeded at a density of 3000 cells/well in 300 µL DMEM, supplemented with 10% fetal bovine serum and 1% antibiotics (penicillin, streptomycin/neomycin) in plates with 96 wells. After 24 h of seeding, the cells were treated with the synthesized Ag/Fe-enhanced TiO_2_ powders and incubated for 72 h. The GSH-Glo Glutathione Assay kit (Promega, WI, USA) was used for oxidative stress assessment. The kit measures the amount of glutathione (GSH) that is produced by cells and converted to oxidized glutathione. The amount of glutathione transformed is directly proportional with the amount of glutathione S-transferase (GST) enzyme that catalyzes the oxidation reaction, which is also linked to the formation of a luminescent luciferin. If the light emission is poor, glutathione production is inhibited and thus oxidative stress is increased, while in the case of an intense light, more glutathione has been synthesized and thus the cell is less stressed.

Initially, 100 µL 1X GSH-Glo Reagent was added, followed by incubation at 37 °C for 30 min. Subsequently, 100 μL Luciferin Detection Reagent was added and incubated at 37 °C for another 15 min. After 15 min, the medium was well homogenized and the plate was read on the luminometer (Microplate Luminometer Centro LB 960, Berthold, Germany) [31].

#### 2.6.3. Evaluation of Cell Morphology and Viability by Fluorescence Microscopy

The biocompatibility of the obtained Ag/Fe-enhanced TiO_2_ powders with AFSC was also evaluated based on fluorescence microscopy using RED CMTPX fluorophore (Thermo Fischer Scientific, Massachusetts, MA, USA), which is a cell tracker for long-term tracing of living cells. The CMTPX tracker was added in cell culture treated with the nanoparticles and the viability and morphology of the AFSC was evaluated after 5 days. The CMTPX fluorophore was added in the culture medium at a final concentration of 5 µM, incubated for 30 min in order to allow the dye penetration into the cells. Next, the AFSC were washed with PBS and visualized by fluorescent microscopy. The photomicrographs were taken with Olympus CKX 41 digital camera driven by CellSense Entry software (Olympus, Tokyo, Japan) [28,31].

## 3. Results and Discussions

**Figure 1:**
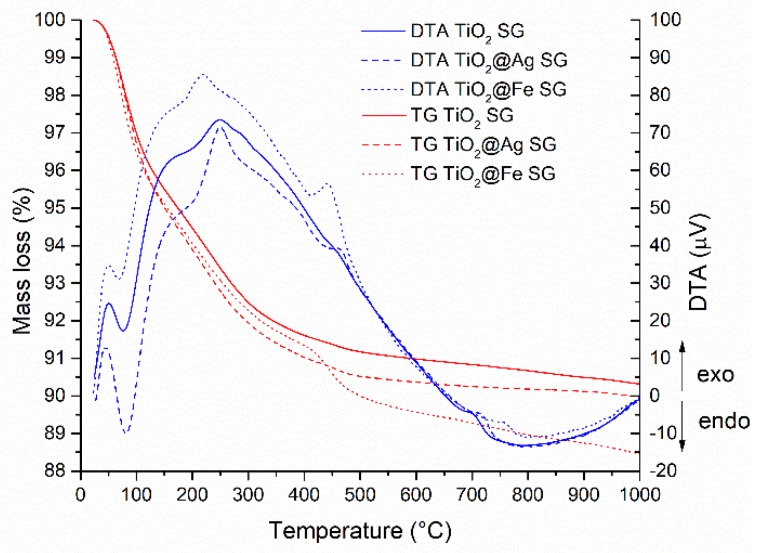
Thermal analysis (DTA and TG) corresponding to the samples obtained by the sol-gel (SG) method.

According to Figure 1, on the ATD curve, around the temperature of 100 °C, an endothermic effect determined by the evaporation of the water used for synthesis was observed and also the evaporation of isopropanol from the sample which is accompanied by a mass loss effect. The exothermic effect correlated with an observed mass loss between 200 and 300 °C is due to the degradation of the excess titanium precursor (TTIP). 

**Figure 2:**
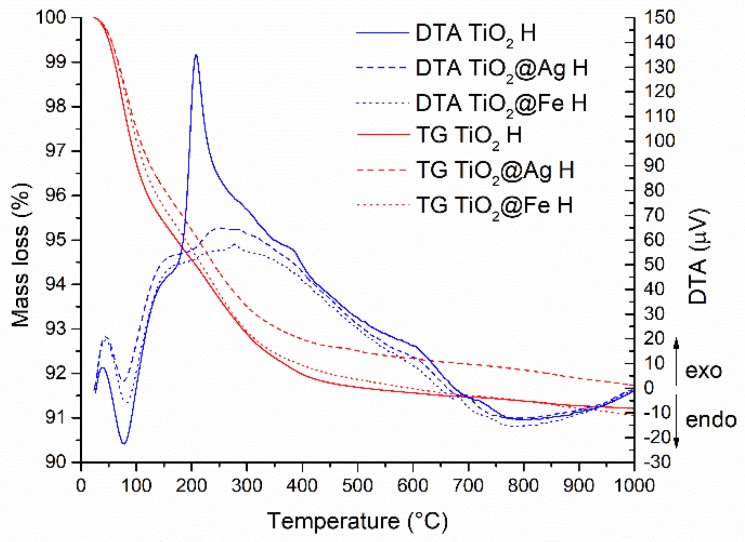
Thermal analysis (DTA and TG) corresponding to the samples obtained by the microwave-assisted hydrothermal (H) method.

In the case of samples obtained by hydrothermal method (Figure 2), on the TG curve, a mass loss (approx. 8%) representative of several thermal effects is determined. Around the temperature of 694 °C for the pure TiO_2_ sample and 718 °C for the enhanced samples, an exothermic effect is observed, without mass loss, which may be due to the anatase-rutile polymorphic transformation.

**Figure 3:**
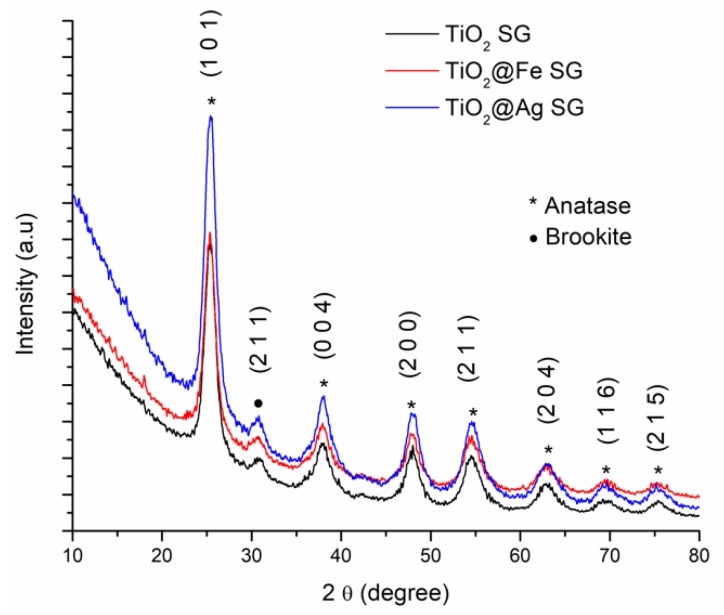
XRD analysis corresponding to the samples obtained by sol-gel (SG) method.

**Figure 4:**
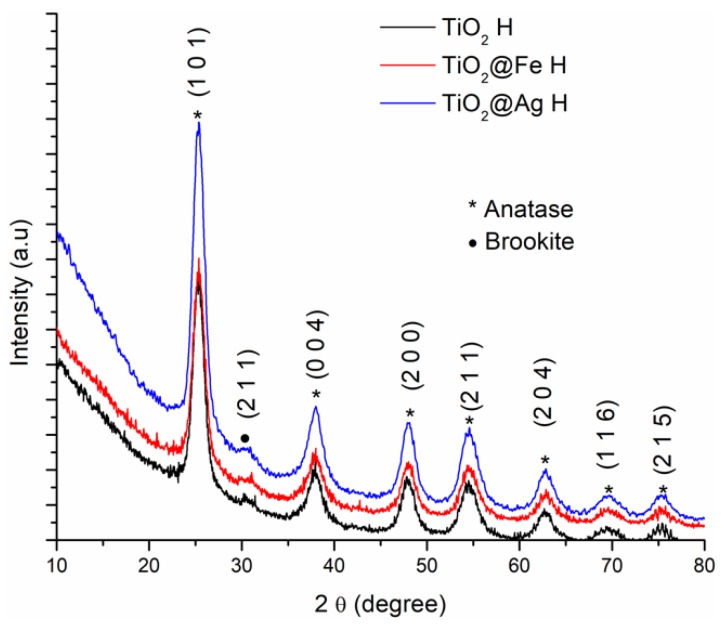
XRD analysis corresponding to the samples obtained by microwave-assisted hydrothermal (H) method.

The XRD results corresponding to the samples obtained by the sol-gel method and the hydrothermal method are shown in Figure 3 and Figure 4.

According to the International Centre for Diffraction Data (ICDD PDF4+) file 04-002-2678, the diffraction peaks identified are specific to the diffraction planes (1 0 1), (0 0 4), (2 0 0), (1 0 5), (2 0 4), (1 1 6), (2 1 5). At the same time, the diffraction peaks specific for the plane (2 1 1), characteristic for brookite was identified (according to the ICDD PDF4+ file 00-002-0514). Table 1 presents the full width at half maximum (FWHM) values corresponding to the peaks associated to most intense plane signal, (1 0 1), along with the calculated crystallite size. 

The FWHM change due to the enhancing effect, this variation is related to the crystallite size being modified by the incorporation of Ag or Fe species in the TiO_2_ crystalline structure. 

**Figure 5:**
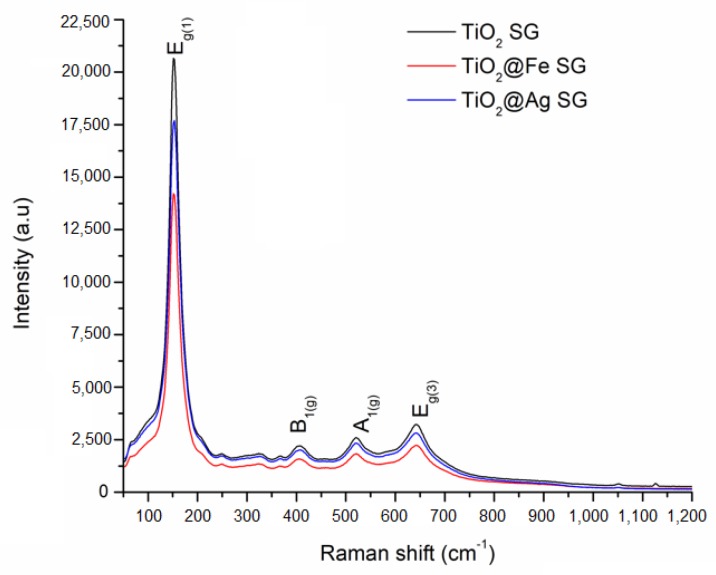
Raman analysis corresponding to the samples obtained by the sol-gel (SG) method.

**Figure 6:**
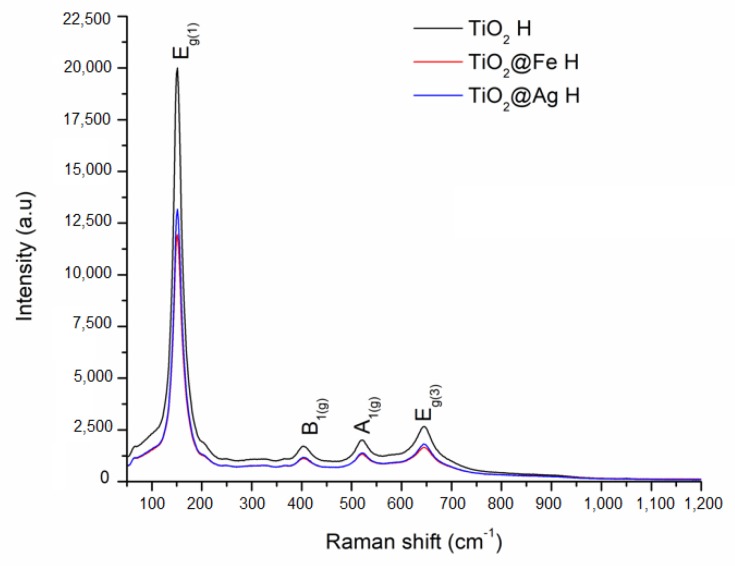
Raman analysis corresponding to the samples obtained by the microwave-assisted hydrothermal (H) method.

The Raman analysis for the samples obtained by sol-gel method and by microwave-assisted hydrothermal method are presented in Figure 5 and Figure 6.

The Raman bands are found at 153, 407, 521, and 642 cm^−1^ for the samples obtained by the sol-gel method and at 151, 404, 521, and 644 cm^−1^ for the samples obtained by the hydrothermal method. The above mentioned vibrational modes are specific to the anatase phase of titania. At the same time, in the case of enhanced samples obtained by both methods of synthesis, a decrease in intensity is observed. This data suggests the presence of Ag/Fe in the structure of the obtained samples. Also, no other bands specific for silver oxide or iron oxide have been identified through the Raman technique [32].

**Figure 7:**
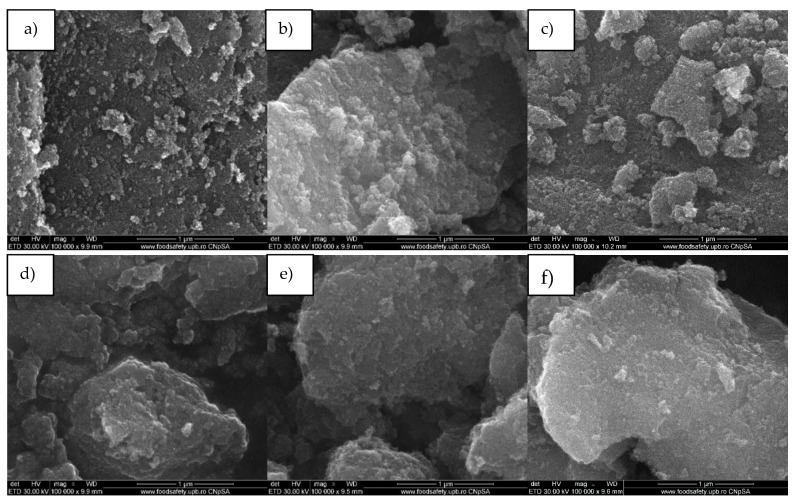
SEM images of nanoparticles (**a**) TiO_2_ SG, (**b**) TiO_2_@Ag SG, (**c**) TiO_2_@Fe SG, (**d**) TiO_2_ H, (**e**)TiO_2_@Ag H, (**f**) TiO_2_@Fe H.

For the pristine and enhanced TiO_2_ samples, SEM analyses (Figure 7) show that the obtained particles have a nanostructured nature. The particles obtained by the hydrothermal method are uniform from a morphological point of view with spheroidal shapes. They are organized in agglomerates with existing dimensions in the micrometric domain and have polyhedral shape.

**Figure 8:**
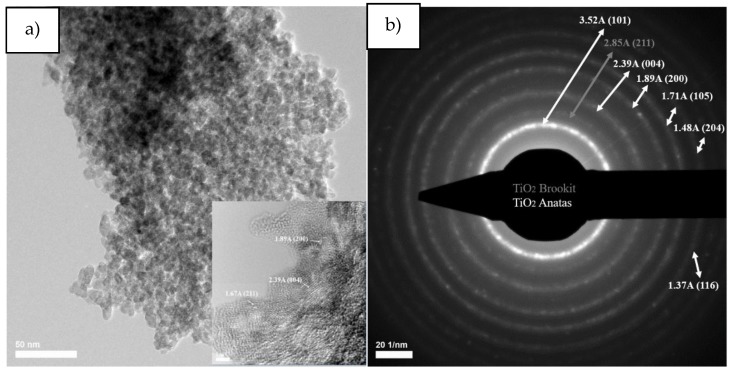

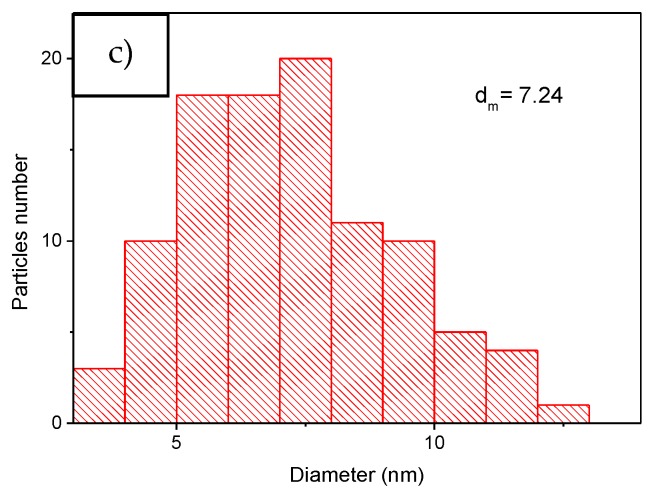
The TEM and HRTEM images (**a**), SAED patterns (**b**) and particle size distribution (**c**) corresponding to TiO_2_ SG sample.

Transmission electron microscopy images for titanium dioxide powder obtained by the sol-gel method (Figure 8) reveal a polyhedral morphology with rounded corners, and selected area electron diffraction reveals diffraction rings specific to the interplanar distances of (1 0 1), (0 0 4), (2 0 0), (1 0 5), (2 0 4), (1 1 6) planes for titanium dioxides in the form of anatase and diffraction rings specific to interplanar distances for the (2 1 1) plane for titanium dioxide in the form of brookite. 

Following the average particle distribution, for the titanium dioxide powder, the average particle size is 7.24 nm, the largest statistics being in the 5–8 nm range, but their size is between 1–13 nm.

**Figure 9:**
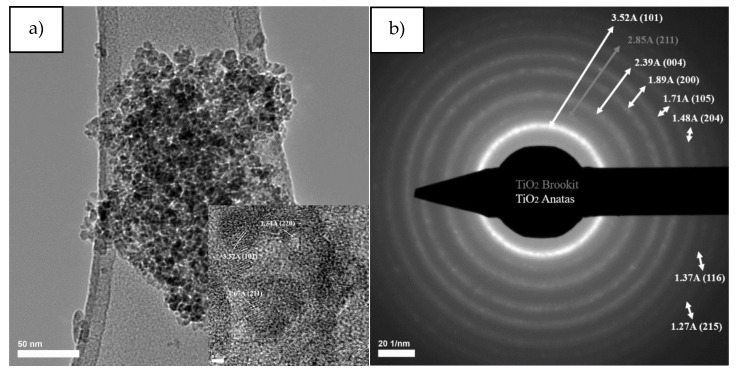

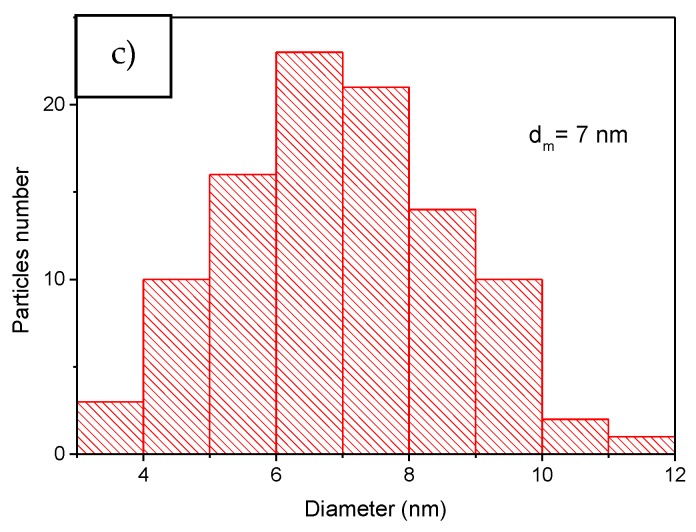
The TEM and HRTEM images (**a**), SAED patterns (**b**) and particle size distribution (**c**) corresponding to TiO_2_@Fe SG sample.

Transmission electron microscopy images for the Fe-enhanced sample (Figure 9) reveal a spheroidal morphology, and electron diffraction on selected area highlights the diffraction rings specific to interplanar distances for the (1 0 1), (0 0 4), (2 0 0), (1 0 5), (2 0 4), (1 1 6), (2 1 5) planes for titanium dioxide in the form of anatase and diffraction ring specific to interplanar distances for the (2 1 1) plane for titanium dioxide in the form of brookite. According to the histogram the average particle size is 7 nm, the largest statistics being in the diameter range of 6–8 nm. 

**Figure 10:**
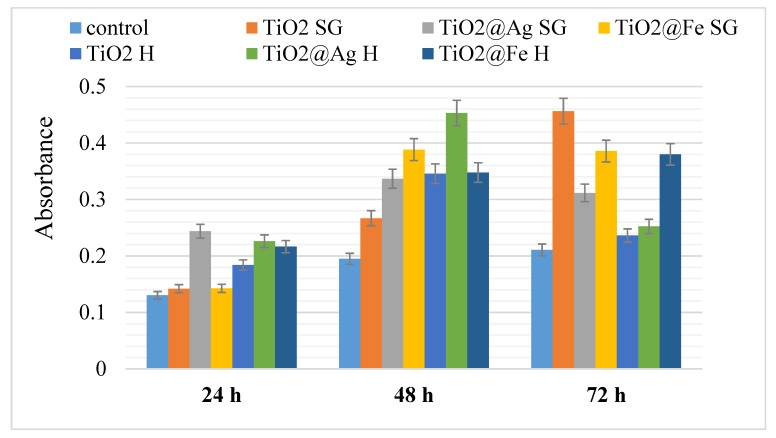
MTT analysis performed on the samples obtained by sol-gel and hydrothermal method.

The results of the MTT analysis show that the samples do not produce cytotoxicity in the tested conditions, all registered absorbance levels being higher than those associated to the control values (the AFSC cells without being in contact with the us obtained powders) at 24, 48 and also 72 h. These results suggest in fact a favoring of AFSC proliferation when in contact with Ag/Fe-enhanced TiO_2_ powders, continuously sustained through a larger period of time—72 h (Figure 10). All results are represented as mean ± standard error, n = 3, * *p* < 0.05.

It is observed that after the first 24 h, the best answer is given by the silver-enhanced samples. This result is most likely due to the antimicrobial activity of silver. After 48 h, all values are increasing compared to control, but after 72 h most values approach the value of the control. The results obtained after 72 h may be a consequence of the release of nanoparticles from cells.

**Figure 11:**
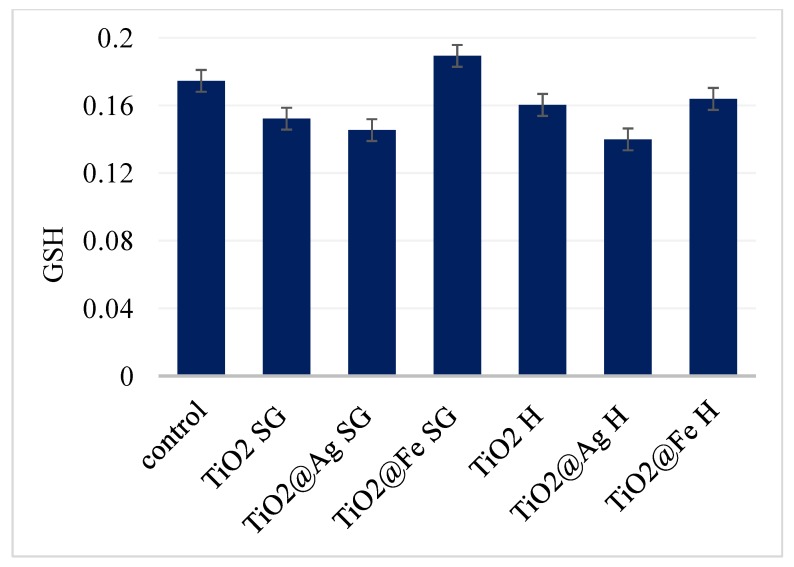
GSH analysis results for samples after 24 h.

In the oxidative stress assessment, the amount of glutathione, a natural antioxidant produced by cells during a normal functioning process, was measured. A high glutathione concentration is associated with the cells being less stressed by the presence of complex nano-systems (unaffected biochemical activity). Therefore, after 24 h it is observed that the level of GSH is slightly lower than in the case of the control sample, but the differences registered are not of significant biological value. Hence we can consider that the samples do not produce significant oxidative stress at the cell level (Figure 11), in accordance with the previously MTT results. 

**Figure 12:**
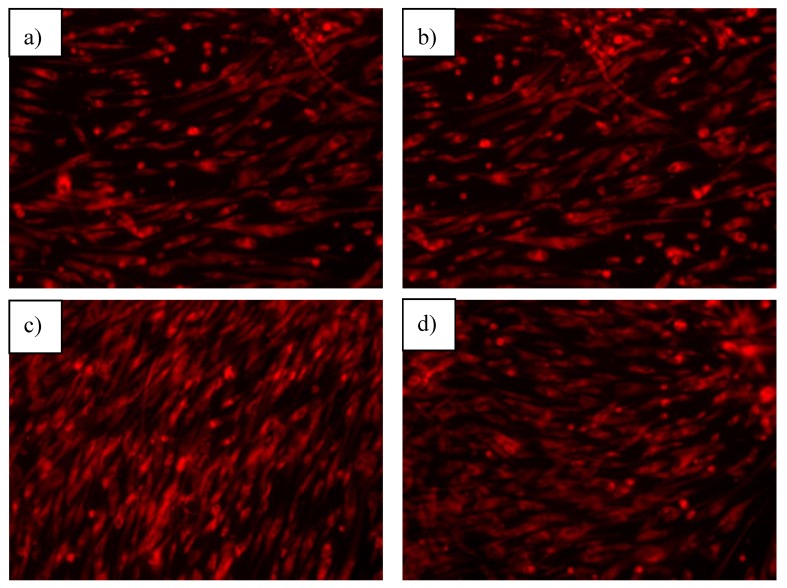

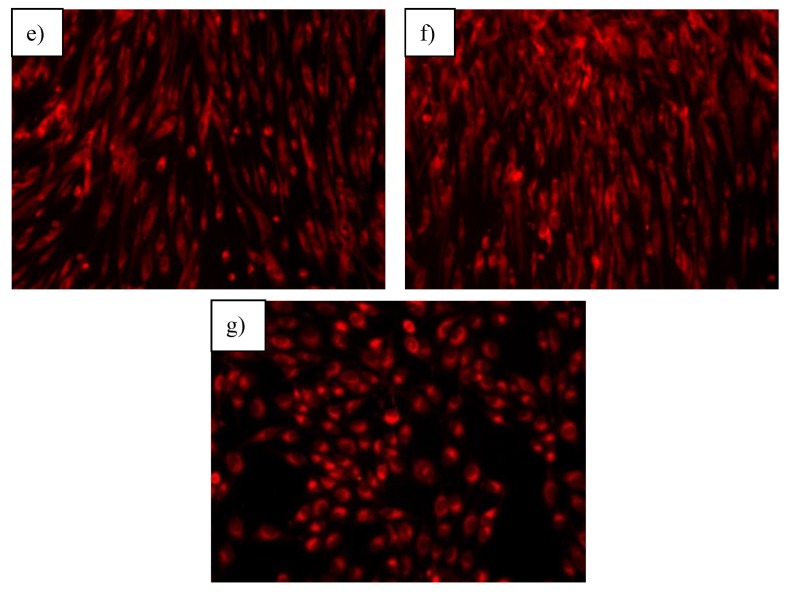
Fluorescence microscopy images of the cells that interacted with the samples (**a**) TiO_2_ SG, (**b**) TiO_2_ H, (**c**) TiO_2_@Ag SG, (**d**)TiO_2_@Ag H, (**e**) TiO_2_@Fe SG, (**f**) TiO_2_@Fe H, (**g**) Control sample.

By the AFSC cells interaction with the sol-gel obtained anatase nanoparticles, it is observed that the morphology of the cytoskeleton changes (Figure 12). The cells become elongated and few retain their spherical shape. In the case of the AFSC cells interaction with the hydrothermal obtained anatase nanoparticles the same change in the morphology of the cytoskeleton is observed, with higher deformation than in the case of the control (especially in the case of non-enhanced samples) and very few cells maintain their initial form. The observed changes are specific to a normal fibroblast-like phenotype, with abundant filopodia, which means that the cells are viable, with an active metabolism and are well attached to the substrate. The occurrence of these pseudopodia is generated by the actin cytoskeleton, a vital protein with high activity in cell interactions and migration [28].

The SPF assessment is a quantitative measurement of the effectiveness of a sunscreen formulation. The in vitro SPF is useful for a screening test during product development, as a prerequisite of the in vivo SPS measure. For the determination of the sun protection factor (SPF) the absorbance values for each sample were taken at wavelengths between 290 to 320 nm (Table 2).

Table 2 highlights that the absorbance values for enhanced samples are much higher than for non-enhanced samples. The SPF of the samples is highly related to the light absorption and their absorption wavelength range. Using Mansur’s formula, it was possible to determine the value of the sun protection factor for each sample. The results are presented in Table 3.

According to Table 3, it can be observed that, in most cases, silver enhancing increases the SPF significantly due to the much higher absorbance value than for non-enhanced samples. The SPF with the highest value is given by a suspension containing sol-gel-derived silver-enhanced anatase nanoparticles. The SPF values obtained for the sol-gel-derived iron-enhanced nanoparticles, as well as hydrothermal-derived silver-enhanced nanoparticles, are also close to the best obtained value. Given that, in regular sunscreen creams, the proportion of nanoparticles is usually 10 wt.%, a reduction of the nanoparticle fraction to half of that value still holds adequate SPF values, which make the above mentioned samples suitable for use in sun protection creams even in 5 wt.%.

## 4. Conclusions

When synthesizing TiO_2_ nanoparticles by wet chemistry (sol-gel versus microwave-assisted hydrothermal synthesis methods) and adding metallic materials, such as Ag or Fe, a series of improvements can be observed, especially from the point of view of their applications. Anatase polymorphic TiO_2_ phase was obtained as a principal crystalline compound in all synthesized samples, with a crystallite size varying from 4 to 8 nm, and a monomodal particles size distribution, with medium diameter range of 6–8 nm. As a prospective compound in sunscreens, the main studied properties are the ability to absorb, scatter and reflect UV rays, quantified by SPF value. In the present study it is shown that the highest SPF value is given by a suspension containing sol-gel-derived silver-enhanced anatase nanoparticles. Corroborating these results with the high biocompatibility proved by the obtained samples for all applied biological characterization methods, we can propose the usage of Ag/Fe-enhanced TiO_2_ nanoparticles as an effective compound in sunscreens.

## Figures and Tables

**Table 1:** FWHM for the characteristic plane (1 0 1) of the anatase phase.

Sample	Plane (1 0 1) Position 2θ (°)	FWHM * (°)	Crystallite Size (nm)
TiO_2_ SG	25.381	1.43	8.06
TiO_2_@Ag SG	25.380	1.47	7.96
TiO_2_@Fe SG	25.310	1.37	5.06
TiO_2_ H	25.346	1.43	9.17
TiO_2_@Ag H	25.407	1.46	5.09
TiO_2_@Fe H	25.352	1.49	4.62

FWHM * full width at half maximum.

**Table 2:** The absorbance of the samples according to the wavelength.

Wavelength (nm)	Absorbance (a.u.)					
	TiO_2_ SG	TiO_2_@Ag SG	TiO_2_@Fe SG	TiO_2_ H	TiO_2_@Ag H	TiO_2_@Fe H
290	2.6984	4.2365	3.9820	1.4026	3.8620	1.6065
295	2.7068	4.2488	3.9984	1.3989	3.8745	1.6087
300	2.7143	4.2627	4.0147	1.3983	3.8859	1.6154
305	2.7273	4.2877	4.0368	1.4045	3.9061	1.6242
310	2.7334	4.2970	4.0511	1.4047	3.9142	1.6257
315	2.7362	4.2895	4.0650	1.4045	3.9251	1.6316
320	2.7355	4.2969	4.0625	1.4063	3.9275	1.6361

**Table 3:** SPF values of suspensions containing 5 wt.% sample.

Sample	TiO_2_ SG	TiO_2_ @Ag SG	TiO_2_ @Fe SG	TiO_2_ H	TiO_2_@Ag H	TiO_2_@Fe H
SPF	27	42	37	14	40	16
